# Impact of the glutamatergic neurotransmission within the A5 region on the cardiorespiratory response evoked from the midbrain dlPAG

**DOI:** 10.1007/s00424-022-02777-6

**Published:** 2022-12-22

**Authors:** M. González-García, L. Carrillo-Franco, C. A. Peinado-Aragonés, A. Díaz-Casares, B. Gago, M. V. López-González, M. S. Dawid-Milner

**Affiliations:** 1 Department of Human Physiology, Faculty of Medicine, University of Malaga, Malaga, Spain; 2 Unit of Neurophysiology of the Autonomic Nervous System (CIMES), University of Malaga, Málaga, Spain; 3 Biomedical Research Institute of Malaga (IBIMA Plataforma BIONAND), Malaga, Spain

**Keywords:** Pontine A5 region, Dorsolateral periaqueductal grey matter (dlPAG), Central cardiorespiratory control, Glutamate, Rat

## Abstract

Stimulation of the dorsolateral periaqueductal grey matter (dlPAG) in rats evokes an active defensive behaviour together with a cardiorespiratory response characterised by tachypnoea, tachycardia and hypertension. The dlPAG neurons involved in these responses are excitatory, presumably glutamatergic, due to the presence of vesicular glutamate transporter VGLUT2 within their axon terminals. Previously, our group described a functional interaction between dlPAG and the pontine A5 region. Accordingly, in the present work, in order to characterize the role of glutamate within this interaction, experiments were carried out in spontaneously breathing anaesthetized rats (sodium pentobarbitone 60 mg/kg i.p., suplemented with 20 mg/kg i.p.). The cardiorespiratory response evoked by electrical stimulation of the dlPAG (1 ms pulses, 20–50 μA, given at 100 Hz, during 5 s) was analysed before and after the microinjection, within the A5 region, of either kynurenic acid (non-specific glutamate receptor antagonist; 5–10 nmol), DAP-5 (NMDA antagonist; 1 pmol), CNQX (non-NMDA antagonist; 1 pmol) or MCPG (metabotropic antagonist; 0,1 nmol). Kynurenic acid decreased the intensity of both the tachypnoea (*p* < 0,001) and tachycardia (*p* < 0,001) induced by dl-PAG stimulation. Blockade of no-NMDA receptors reduced the increase of respiratory frequency, heart rate and pressor response to dl-PAG stimulation (*p* < 0,01, *p* < 0,001, *p* < 0,05 respectively). Blockade of either NMDA or metabotropic receptors reduced the dlPAG-evoked tachycardia and pressor response (*p* < 0,01; *p* < 0,05 respectively). These results suggest a neuromodulatory role for A5 region via glutamate neurotransmission of the dlPAG-evoked cardiorespiratory response, confirming the role of the ventrolateral pons in the neuronal circuits involved in respiratory and heart rate control.

## Introduction

The midbrain periaqueductal gray matter (PAG) is an anatomical and functional interface between the forebrain and the pons and its main function is to integrate active and passive behavioural responses to internal or external stimuli [14, 16]. Anatomically, it is structured in four longitudinal columns, which are named: dorsomedial (dm), dorsolateral (dl), lateral (l) and ventrolateral (vl). Each column has different properties, not only functional, but also regarding connectivity and chemical properties [2, 34].

Due to its afferent-efferent connections, the PAG modulates several patterns of cardiorespiratory, motor and nociceptive responses. The main afferents are those arriving from the prefrontal cortex, amygdala, hypothalamus and nociceptive pathways, which are integrated and project from the dlPAG to different nuclei of the pons and medulla involved in autonomic control. Thus, PAG allows the modulation of a variety of autonomic responses that depend on the type of stress and the individual's own subjective perception of the supposed threat or stressor [8, 14, 32, 33]. Other PAG functions include thermoregulation, wakefulness and sleep mechanisms, modulation of neuropathic pain, urination and vocalization [6, 11, 15, 30]. Clinically, it is known to participate in different neurodegenerative processes such as Alzheimer's disease and multisystem atrophy [1, 24].

Recently, our research group has shown a functional connection between the dlPAG and the pontine A5 region [19]. Muscimol (GABAergic agonist) microinjections within the A5 region produced a decrease in the magnitude of the tachycardic and tachypnoeic responses evoked by dlPAG electrical stimulation. Moreover, applying single unit extracellular neuronal recordings, we have observed that several A5 neurons modified their spontaneous frequency of discharge during dlPAG stimulation. These results provided the first electrophysiological evidence of a direct interaction between dlPAG and A5 neurons.

Both regions, dlPAG and A5, have been largely studied. It is well established that their activation produces an increase in sympathetic tone. In the case of dlPAG, it includes an increase in cardiorespiratory parameters (blood pressure, heart rate and respiratory frequency) [3, 9], whereas the activation of the A5 region produces similar cardiovascular changes but associated with a decrease in respiratory frequency [4, 5, 22, 31].

Several authors have shown that both regions, dlPAG and A5, work together with the hypothalamic defence area located in the dorsomedial hypothalamic region and perifornical area (DMH-PeF) [6, 21]. The cardiovascular component of the defence response evoked from DMH-PeF is also mediated by glutamatergic receptors within the A5 region [20].

As dlPAG efferent projections are known to be excitatory, presenting vesicular glutamate transporter type 2 (VGLUT-2) immunopositive ( +) axon terminals [8, 17], and having demonstrated the functional relationship between the A5 region and dlPAG, we hypothesised a possible role of glutamate in the interaction between both regions. Both ionotropic, NMDA (NR1-NR2D), non-NMDA (AMPA and kainite) and metabotropic (mGlu I, II and III) NMDA receptors have been demonstrated to be expressed within the A5 region [10, 29, 35].

Therefore, the main objective of this work was to test the possible role of A5 glutamate receptors in the cardiorespiratory response evoked by dlPAG stimulation. To this end, we have blocked A5 region glutamatergic receptors with microinjections of kynurenic acid, a broad-spectrum glutamate antagonist. Once this part was done, the study was completed by selectively blocking the different subtypes of glutamate receptors by microinjections of DAP5 (non-competitive ionotropic NMDA antagonist), CNQX (competitive ionotropic non-NMDA antagonist) and MCPG (non-selective metabotropic antagonist type I and II).

## Methods

These studies were undertaken with the same protocols previously described by our group [19, 20].

### Animals and housing

Studies were performed on 50 male SPF Sprague–Dawley rats (250–350 g; Charles River, Barcelona, Spain). Animals were housed six per cage in a temperature-controlled room (22–24º C) and maintained on a 12:12 h light / dark cycle (light at 8:00 am) in the Animal House of the University of Malaga. Food and water were available ad libitum.

### General procedures

The surgical procedures were similar to those of previous studies [19, 20] and were performed under anaesthesia with sodium pentobarbitone (60 mg kg^−1^ i.p, initial dose, supplemented as necessary with 2 mg kg^−1^ i.v.). The femoral artery and vein were cannulated for the measurement of arterial blood pressure and for the administration of drugs, respectively. In order to measure respiratory flow (Fleish pneumotachograph) and pleural pressure (air filled catheter), both trachea and oesophagus were cannulated. The animals breathed spontaneously a mixture of humidified O_2_ enriched room air. End tidal CO_2_ was monitored during the experiment with a fast response CO_2_ analyser (ADC FM1, The Analytical Development Co. Ltd.,Great Amwell, UK), with values ranged from 3 to 5%. During procedures, the animals were kept al 37ºC using a heated surgical table and a servo-controlled heating pad (rectal temperature) to prevent hypothermia. Anaesthesia was monitored during the procedures and throughout the experiment by both measuring under resting conditions possible changes in cardiovascular variables and assessing the absence of a significant withdrawal reflex to pinching a paw. The head of the animals was fixed in a stereotaxic frame (upper incisor bar 3.3 mm below interaural line).

#### dlPAG–A5 interaction: stimulation experiments

Two burr holes were drilled into the skull of each animal to access to the right dlPAG and the A5 region through the cerebellum. The right dlPAG was stimulated with 1 ms pulses, 30–50 µA given at 100 Hz for 5 s by positioning a concentric bipolar electrode (NE-100; Rhodes Medical Electrodes, Summerland, CA, USA). The stereotaxic coordinates to locate the right dlPAG were from -4.0 mm caudal to bregma, 0.6 lateral to midline and 4.5 to 5 mm depth from the surface of the calota (approaching with an angle of 30º).

Glutamate receptors of the ipsilateral A5 region to the stimulated dlPAG were pharmacologically blocked by means of either kynurenic acid 100 mM, DAP5 2 mM, CNQX 1 M or MCPG 100 mM microinjected through a stereotaxically positioned single glass micropipette. A solution of sodium-phosphate buffer saline (PBS, pH 7.4 ± 0.1) with 0.05% Evans blue was used to dissolve all drugs. Evans blue served to mark microinjection sites. Microinjections of PBS-Evans blue alone were used for control purposes. The micropipette was positioned stereotaxically according to the following coordinates: from interaural line to -1.6 mm, 2–3 mm lateral to midline and 1 to -0.5 mm depth to interaural line.

Microinjection volumes of 50 nl were programmed with a micropump controller (Ultra Micro Pump II, Micro 4; World Precision Instruments, Inc., Sarasota, FL, USA), driving 0.5 µl microsyringes attached to the micropipette. Only one microinjection was delivered in each animal.

Electrical lesions (250 μA DC for 20 s) or Evans blue serve to locate dlPAG and A5 region stimulation/microinjections sites, respectively. Brains were perfused with 10% formal saline, serially sectioned (50 µm) at the level of the midbrain and the pons. The midbrain and pons were counter stained with Neutral Red.

The protocol that was used in each group of experiments was the following:

- Study of cardiorespiratory parameters at rest (pre-drug).
- Study of cardiorespiratory changes during dlPAG electrical stimulation (pre-drug stimulation).
- Study of cardiorespiratory parameters at rest 4 min after the injection of PBS (50 nl, pH 7.4 ± 0.1, 5 s duration), kynurenic acid (50 nl, 5 nmol, pH 7.4 ± 0.1, 5 s duration), DAP5 (50 nl, 0,1 nmol, pH 7.4 ± 0.1, 5 s duration), CNQX (50 nl, 50 nmol, pH 7.4 ± 0.1, 5 s duration) or MCPG (50 nl, 5 nmol, pH 7.4 ± 0.1, 5 s duration) into the A5 region (post-drug).
- Study of cardiorespiratory changes during dlPAG electrical stimulation after PBS, kynurenic acid, DAP5, CNQX or MCPG was delivered into A5 region (post-drug stimulation).

Thus, 4 measurements (pre-drug; pre-drug stimulation; post-drug; post-drug stimulation) in each of the 5 groups (PBS, kynurenic acid, DAP5, CNQX or MCPG) were obtained.

In each animal airflow, respiratory volume, pleural pressure (as an index of inspiratory activity) and arterial pressure were monitored and stored on PC. Measurements were made of inspiratory time, expiratory time, instantaneous respiratory frequency, pleural pressure, mean blood arterial pressure and instantaneous heart rate. In all experiments baseline values for mean arterial blood pressure, heart rate and respiratory parameters were measured immediately prior to dlPAG electrical stimulation. Changes in mean arterial blood pressure or heart rate were assessed by measuring the peak rise in blood pressure or heart rate observed during the 5 s stimulation of the dlPAG. Stimulus-evoked changes in respiratory parameters were measured as the average response observed during the 5 s stimulation of the dlPAG. Recordings were taken for 3 min, starting 30 s before the beginning of a stimulus. The 3 min window used for data analysis allowed a complete recovery of the evoked response. The offline analysis was done using LabChartPro (PowerLab System, ADInstruments®/ LabChart Software, version 8.0, Sydney, Australia).

### Statistical procedures

All data are expressed as mean ± SEM. For statistical comparisons, once the statistical normality (Kolmogorov–Smirnov test) and the homoscedasticity (Bartlett's test) of the data were verified, a paired-sample test was applied to compare the control with the evoked response period for each animal. One-way ANOVA was used to compare different groups of animals. At each time point, *p* < 0.05 was regarded as significant. Only data from animals in which the histology showed that the microelectrodes were positioned within the dlPAG and the A5 region were used for statistical procedures.

## Results

### I- dlPAG cardiorespiratory response

**Fig. 1:**
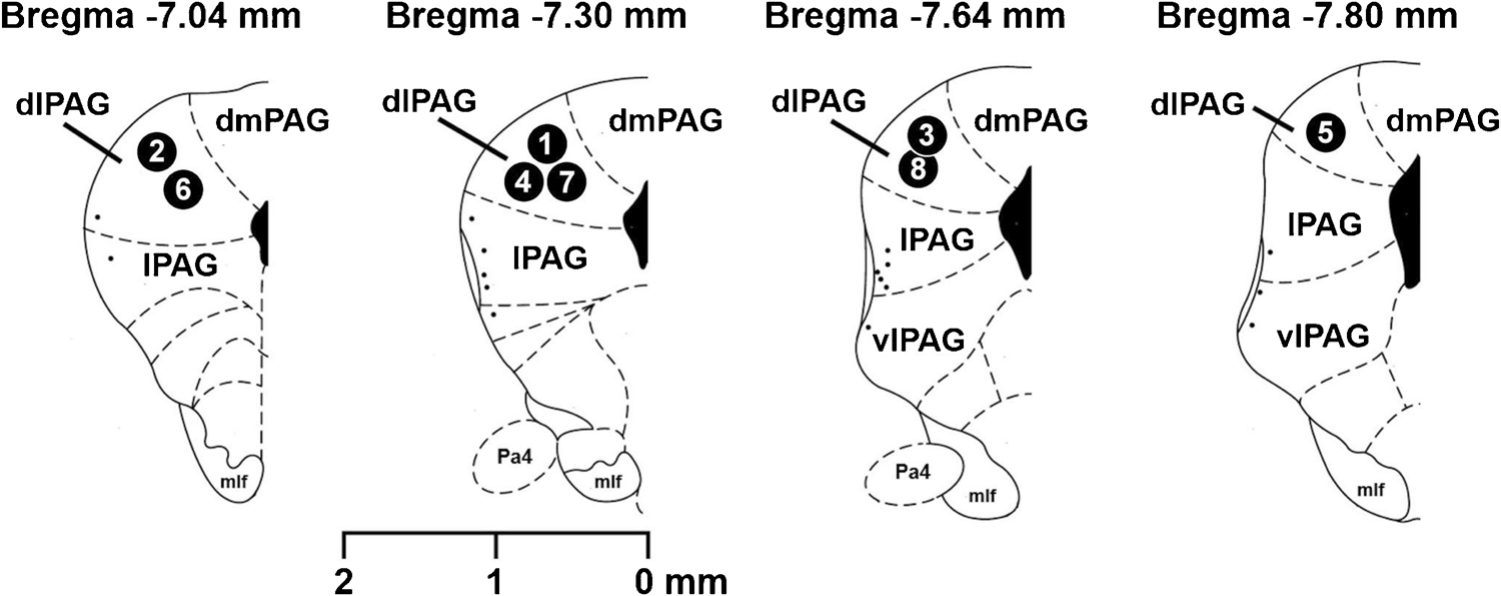
Semi-schematic line drawings of coronal sections through the dlPAG from rostral (left) to caudal (right) areas, showing the sites where electrical stimulations were applied. Black numbered circles show the location of electrical stimulation in animals which received kynurenic microinjections within the A5 region. dmPAG, dorsomedial periaqueductal grey; dlPAG, dorso-lateral periaqueductal grey; lPAG, lateral periaqueductal grey; vlPAG, ventro-lateral periaqueductal grey. (mlf) medial longitudinal fasciculus. (Pa4) paratrochlear nucleus

**Fig. 2:**
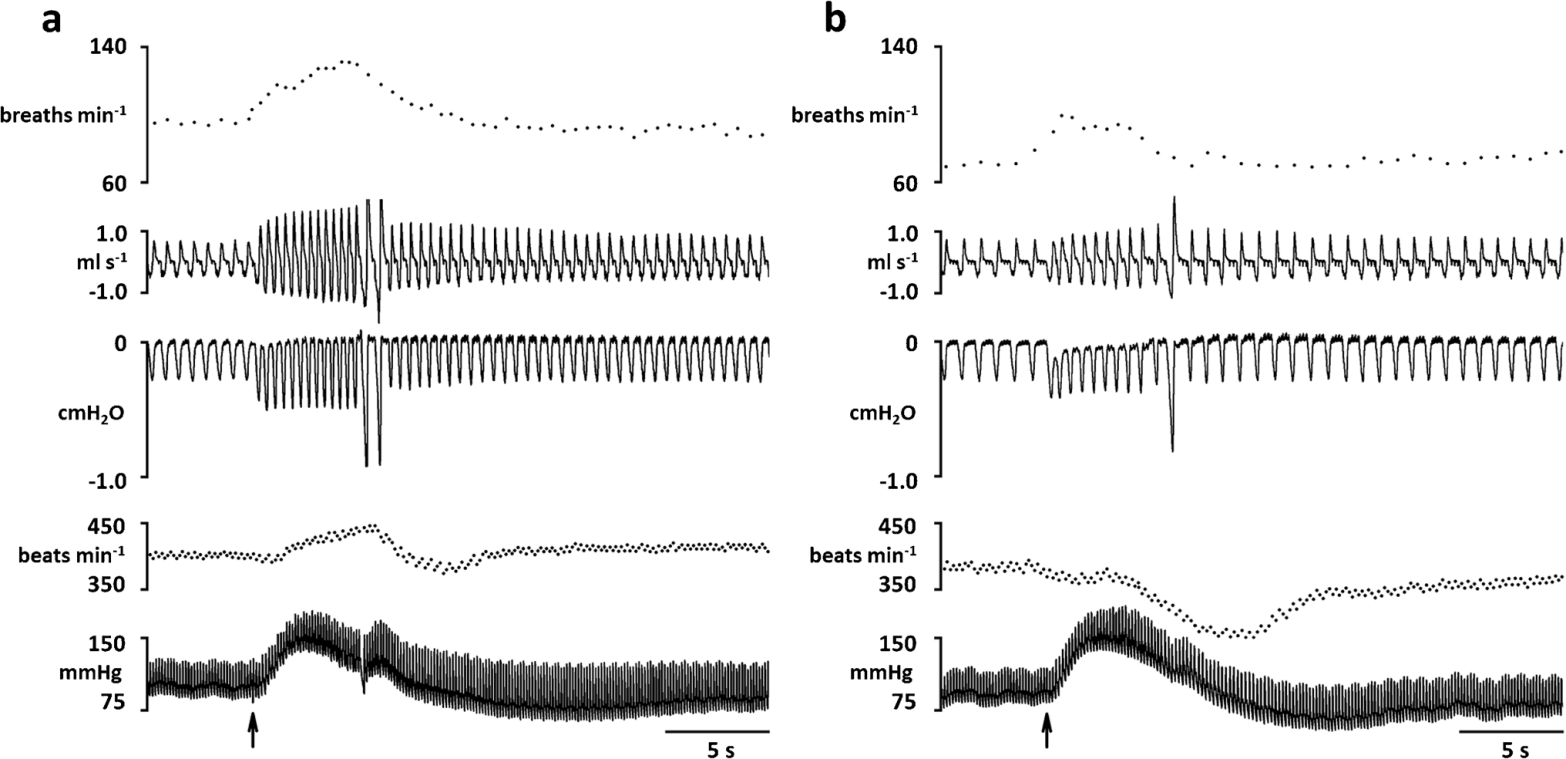
Instantaneous respiratory rate (upper trace, breaths min^−1^), respiratory flow (ml/s), pleural pressure (cm H_2_O), instantaneous heart rate (beats min^−1^) and blood pressure (mmHg) in a spontaneously breathing rat showing the cardiorespiratory response evoked to dlPAG stimulation before (**a**) and after the microinjection of kynurenic in the A5 region (50 nl over 5 s) (**b**). Black arrow shows the onset of the dlPAG electrical stimulation (5 s)

In all animals (*n* = 36) dlPAG electrical stimulation (pre-drug stimulation) elicited a cardiorespiratory response characterized by tachypnoea (*p* < 0,001), an increase in inspiratory activity (*p* < 0,001) and a pressor response (*p* < 0,001) accompanied with tachycardia (*p* < 0,001) (Figs. 1 and 2a).

### II- Glutamate broad antagonist: PBS vs kynurenic acid

#### PBS microinjection within A5 region

**Fig. 3:**
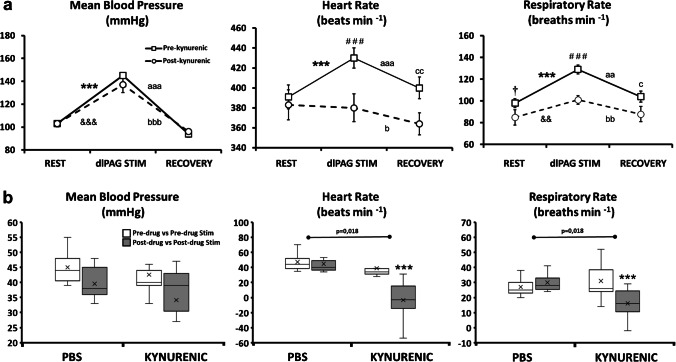
**a** Changes of mean blood pressure, heart rate and respiratory rate before (rest), during (dlPAG stimulation) and 5 min after (recovery) dlPAG stimulation (before (pre-kynurenic, empty square) and after microinjection of kynurenic (post-kynurenic, empty circle) within the A5 region). Data expressed as mean ± SEM. ***p<0.001, pre-drug vs pre-drug stimulation; &&*p*<0.01, &&&*p*<0.001, post-drug vs post-drug stimulation; †*p*<0.05, pre-drug vs post-drug; ###p<0.001, pre-drug stimulation vs post-drug stimulation; aa*p*<0.01, aaa*p*<0.001, pre-drug stimulation vs recovery; b*p*<0.05, bb*p*<0.01, bbb*p*<0.001, post-drug stimulation vs recovery; c*p*<0.05, cc*p*<0.01, pre-drug recovery vs post-drug recovery. **b** Box-Whisker graphs showing the differences between the delta cardiorespiratory responses after PBS or kynurenic microinjections within the A5 region during dlPAG stimulation (mean blood pressure, heart rate and respiratory rate responses) (before (pre-drug vs pre-drug stim; empty box) and after (post-drug vs post-drug stim; filled box) microinjections of PBS or kynurenic within the A5 region). ***p<0.001

Microinjections of PBS into the A5 region (*n* = 7) did not produce changes neither in resting cardiorespiratory parameters (pre-drug vs post-drug) nor in the amplitude of the dlPAG evoked cardiorespiratory response (post-drug vs post-drug stimulation) compared with control situation (pre-drug vs pre-drug stimulation) (Fig. 3b; Table 1).

**Table 1:** Respiratory and cardiovascular changes before electrical stimulation of dlPAG (Pre-drug), during electrical stimulation of dlPAG (Pre-drug stim), 4 min after PBS, kynurenic acid, DAP-5, CNQX or MCPG microinjections within the A5 region (Post-drug) and during dlPAG electrical stimulation after PBS, kynurenic acid, DAP-5, CNQX or MCPG microinjections within the A5 region (Post-drug stim)

PBS	Pre-drug	Pre-drug Stimulation	Post-drug	Post-drug Stimulation
A5 (*n=7)*				
Ti (s)	0.203 ± 0.01	0.194 ± 0.01	0.205 ± 0.01	0.197 ± 0.01
Te (s)	0.343 ± 0.02	0.238 ± 0.02***	0.352 ± 0.03	0.242 ± 0.03^&&&^
PP (cm H_2_O)	0.369 ± 0.03	0.509 ± 0.04*	0.393 ± 0.02	0.506 ± 0.03^&^
RR (rpm)	111 ± 6	138 ± 7***	109 ± 5	139 ± 8^&&&^
BP (mmHg)	102 ± 2	147 ± 4***	104 ± 4	144 ± 3^&&&^
HR (bpm)	339 ± 9	386 ± 12***	342 ± 11	387 ± 10^&&&^
*Kynurenic acid*	Pre-drug	Pre-drug Stimulation	Post-drug	Post-drug Stimulation
A5 (*n=8)*				
Ti (s)	0.258 ± 0.01	0.215 ± 0.01*	0.253 ± 0.01	0.245 ± 0.02
Te (s)	0.362 ± 0.02	0.255 ± 0.02**	0.480 ± 0.04^††^	0.350 ± 0.02 ^& ##^
PP (cm H_2_O)	0.302 ± 0.03	0.519 ± 0.03**	0.388 ± 0.02	0.516 ± 0.04^&^
RR (rpm)	98 ± 4	129 ± 4***	85 ± 7^†^	101 ± 4 ^&& ###^
BP (mm Hg)	103 ± 3	145 ± 2***	103 ± 2	137 ± 7 ^&&&^
HR (bpm)	391 ± 12	430 ± 10***	383 ± 15	380 ± 14^###^
*DAP-5*	Pre-drug	Pre-drug Stimulation	Post-drug	Post-drug Stimulation
A5 (*n=7)*				
Ti (s)	0.283 ± 0.02	0.235 ± 0.01*	0.289 ± 0.04	0.250 ± 0.01^&^
Te (s)	0.386 ± 0.02	0.266 ± 0.02***	0.398 ± 0.03	0.290 ± 0.02^&&^
PP (cm H_2_O)	0.365 ± 0.03	0.591 ± 0.04***	0.356 ± 0.02	0.569 ± 0.04^&&&^
RR (rpm)	92 ± 5	121 ± 4***	90 ± 4	112 ± 2^&&^
BP (mm Hg)	104 ± 2	174 ± 3***	113 ± 4	174 ± 6^&&&^
HR (bpm)	362 ± 17	403 ± 11***	369 ± 12	377 ± 13^#^
*CNQX*	Pre-drug	Pre-drug Stimulation	Post-drug	Post-drug Stimulation
A5 (*n=7)*				
Ti (s)	0.202 ± 0.01	0.176 ± 0.01**	0.191 ± 0.01	0.189 ± 0.01
Te (s)	0.358 ± 0.02	0.262 ± 0.02***	0.434 ± 0.04	0.313 ± 0.02^&&& #^
PP (cm H_2_O)	0.380 ± 0.02	0.631 ± 0.05***	0.403 ± 0.02	0.648 ± 0.05^&&&^
RR (rpm)	109 ± 5	141 ± 8***	98 ± 5	122 ± 6^&&& #^
BP (mm Hg)	109 ± 2	160 ± 5***	128 ± 4^††^	172 ± 4^&&& ##^
HR (bpm)	323 ± 10	360 ± 7***	350 ± 8^††^	368 ± 6^&&^
*MCPG*	Pre-drug	Pre-drug Stimulation	Post-drug	Post-drug Stimulation
A5 (*n=7)*				
Ti (s)	0.255 ± 0.01	0.204 ± 0.01**	0.245 ± 0.01	0.227 ± 0.01^#^
Te (s)	0.428 ± 0.04	0.337 ± 0.02***	0.468 ± 0.05	0.320 ± 0.03^&&^
PP (cm H_2_O)	0.333 ± 0.02	0.519 ± 0.02***	0.319 ± 0.02	0.505 ± 0.02^&&&^
RR (rpm)	91 ± 6	115 ± 6**	87 ± 7	112 ± 6^&&^
BP (mmHg)	105 ± 3	166 ± 8***	116 ± 8	164 ± 11^&&&^
HR (bpm)	336 ± 12	377 ± 13***	340 ± 14	359 ± 17^&&^

Data are expressed as mean ± SEM. *p < 0.05, **p < 0.01, ***p < 0.001 Pre-drug vs Pre-drug Stimulation; ^&^*p* < 0.05, ^&&^*p* < 0.01, ^&&&^*p* < 0.001 Post-drug vs Post-drug Stimulation; ^†^*p* < 0.05, ^††^*p* < 0.01 Pre-drug vs Post-drug; ^#^*p* < 0.05, ^##^*p* < 0.01, ^###^*p* < 0.001 Pre-drug Stimulation vs Post-drug Stimulation. Abbreviations: Inspiratory time (Ti). Expiratory time (Te). Respiratory Rate (RR). Pleural Pressure (PP). Blood Pressure (BP). Heart Rate (HR)

#### Kynurenic acid microinjections within A5 region

**Fig. 4:**
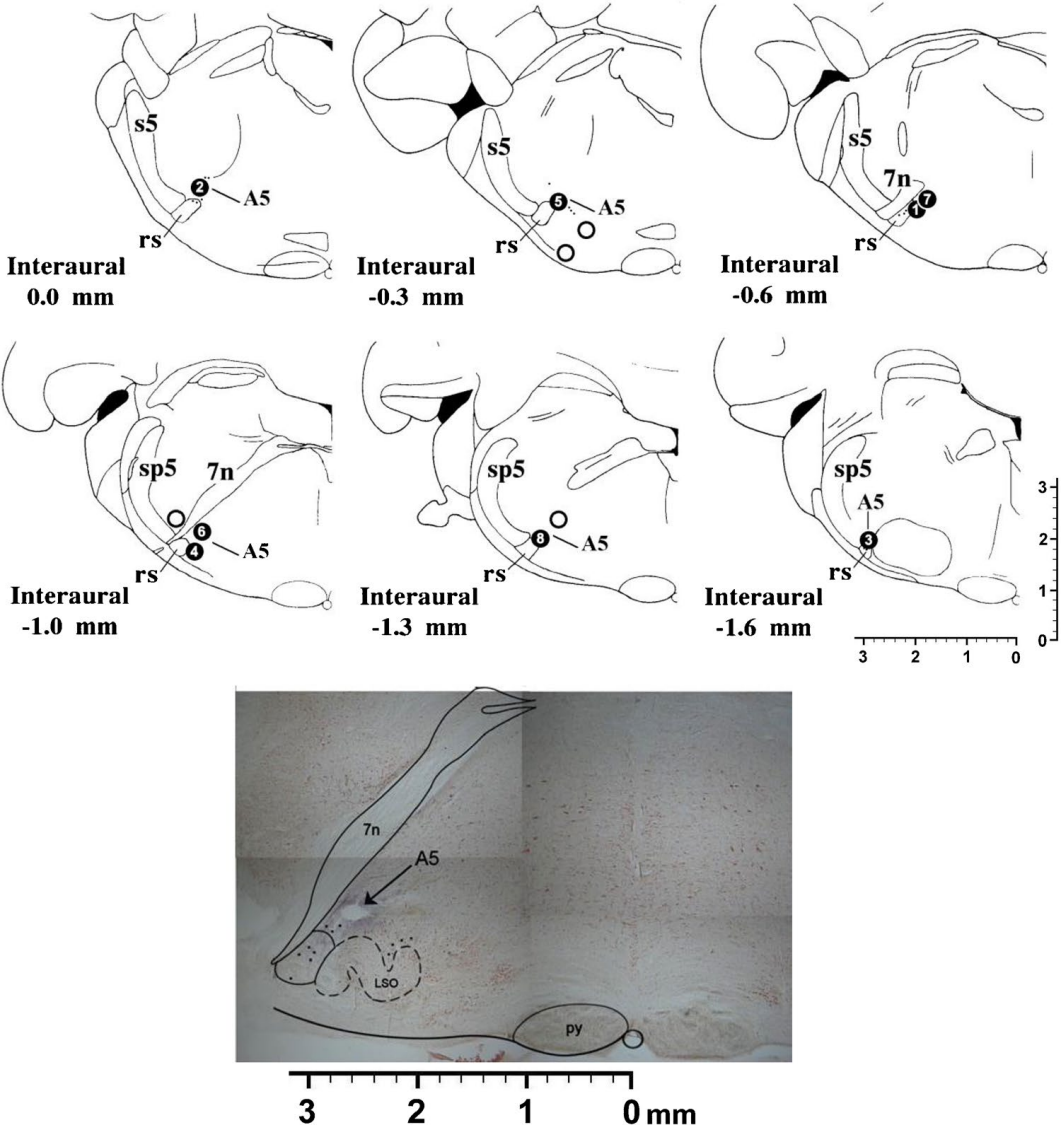
Semi-schematic line drawings of coronal sections through the A5 region from rostral (top left) to caudal (bottom right), showing the sites where microinjections were applied. Filled black numbered circles show the location of kynurenic microinjections within A5 region. Empty circles show locations in which kynurenic was delivered to sites outside the A5 region. Each circle corresponds to one animal. Composed photomicrograph of a coronal section through the A5 showing location of stimulation. s5, sensory root trigeminal nerve; sp5, spinal trigeminal tract; rs, rubrospinal tract; 7n, facial nerve or its root

Comparing resting situations (pre-drug vs post-drug), kynurenic acid microinjection in A5 region (*n* = 8) (Fig. 4) produced a significant decrease in respiratory rate (*p* < 0.05) due to a significant increase in expiratory time (*p* < 0.01) (Table 1). Neither the cardiovascular component nor inspiratory time or pleural pressure were significantly modified (Figs. 2b and 3a; Table 1).

##### *Respiratory response to dlPAG stimulation*

**Fig. 5:**
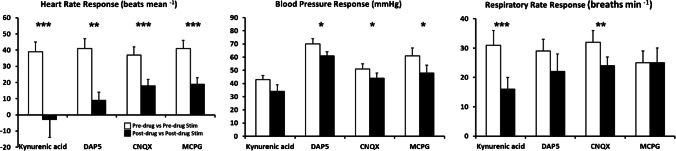
Changes in the intensity of the cardiorespiratory response evoked on dlPAG stimulation  (respiratory rate response, blood pressure response and heart rate response) before (pre-drug vs pre-drug stimulation (empty bar)) and after (post-drug vs post-drug stimulation (filled bar)) microinjection of kynurenic acid, DAP5, CNQX and MCPG respectively within the A5 region. *p<0.05; **p<0.01; ***p<0.001

The dlPAG stimulation performed after kynurenic acid microinjection produces a significant increase in respiratory rate (*p* < 0,01) (post-drug vs post-drug stimulation) but, compared with control situation (pre-drug vs pre-drug stimulation), the amplitude of the response was decreased (*p* < 0,001) (Figs. 2b, 3a and 5; Table 1).

##### *Cardiovascular response to dlPAG stimulation*

After kynurenic acid microinjection dlPAG stimulation evoked an increase in blood pressure (*p* < 0,001) (post-drug vs post-drug stimulation). Besides, the tachycardia observed at pre-drug stimulation was abolished by the kynurenic acid microinjection within the A5 region (*p* < 0,001; pre-drug stimulation vs post-drug stimulation) (Table 1). The amplitude of the heart rate component of the cardiovascular response evoked by electrical stimulation of the dlPAG (post-drug vs post-drug stimulation) was substantially modified after microinjection of kynurenic acid within the A5 region compared with control situation (pre-drug vs pre-drug stimulation) (*p* < 0,001). No changes in the amplitude of the blood pressure response were observed (Figs. 2b, 3a and 5; Table 1).

##### *Other results*

It should be noted that, initially, kynurenic was administered bilaterally in a group of 5 animals, but it caused a gradual respiratory depression. Thus, these animals were not included for experimental procedures.

Four other animals were microinjected outside the area of study (see Fig. 4). In the first one, the microinjection was located above of the exit of the facial nerve (7n), and a slight decrease in blood pressure and a slight increase in respiratory rate as a consequence of dlPAG stimulation after microinjection of kynurenic acid was observed. Another animal had a decrease in blood pressure and heart rate after dlPAG stimulation, with the location of the microinjection in the superior lateral olive. Two animals showed no changes after dlPAG stimulation compare with control stimulation. The location of the microinjection sites were in the area of the superior paraolivary nucleus (SPO) and superior lateral olive (LSO); and in the accessory nucleus abducens and accessory facial nucleus (Acs 6/7).

#### PBS vs kynurenic acid A5 microinjections

When comparing the differences at the cardiorespiratory responses between PBS and kynurenic acid microinjected animals during dlPAG stimulation, higher values in both delta heart rate (-2,4 ± 0,4 to -42,3 ± 8,5; *p* = 0,018) and delta respiratory rate (3 ± 0,3 to -15 ± 2,5; *p* = 0,018) responses were observed (Fig. 3b). No significant changes were obtained comparing the delta blood pressure response (-5,4 ± 0,4 to -8,4 ± 4,6) (Fig. 3b).

### III- Glutamate specific antagonists

#### Ionotropic NMDA antagonists

##### *Cardiorespiratory effect produced by ionotropic NMDA glutamatergic antagonist in the A5 region: DAP5 microinjection*

DAP5 microinjected within the A5 region (*n* = 7) did not produce significant changes in any resting cardiorespiratory parameters (pre-drug vs post-drug) (Table 1).

##### *Role of A5 DAP5 microinjections on the respiratory response to dlPAG stimulation*

The dlPAG stimulation performed after blockade of A5 region NMDA receptors with DAP5 microinjection (post-drug vs post-drug stimulation) produces a significant increase in both respiratory rate (*p* < 0,01) and pleural pressure (*p* < 0,001) (Table 1) but, compared with control situation (pre-drug vs pre-drug stimulation), the amplitude of the respiratory response was similar (Figs. 5).

##### *Role of A5 DAP5 microinjections on the cardiovascular response to dlPAG stimulation*

The dlPAG stimulation after DAP5 microinjection produced an increase in the blood pressure response (*p* < 0,001) (post-drug vs post-drug stimulation), similar to that produced during pre-drug stimulation. The heart rate component of the cardiovascular response was not modified by the dlPAG stimulation (post-drug vs post-drug stimulation), contrary to the tachycardia observed at pre-drug stimulation (*p* < 0,05) (Table 1).

When comparing the amplitude of the changes to dlPAG stimulation (post-drug vs post-drug stimulation) with control situation (pre-drug vs pre-drug stimulation), a significant decrease in heart rate (*p* < 0.01) and blood pressure (*p* < 0.05) values were observed (Fig. 5).

##### *Other results*

In two animals, the microinjections were delivered outside the area of study. In the first one, there was a slight decrease in blood pressure after stimulation of the dlPAG compare with control situation. The location was between the areas of the main trigeminal sensory nucleus ventrolateral part—trigeminal spinal tract (Pr5VL-Sp5). In the other animal, the cardiorespiratory response remained unchanged after dlPAG stimulation compared with control situation. The location of the microinjection site was in the area of the SPO and LSO.

#### Ionotropic non-NMDA antagonists

##### *Cardiorespiratory effect produced by ionotropic non-NMDA glutamatergic antagonist in the A5 region: CNQX microinjection*

CNQX microinjected into the A5 region (*n* = 7) produced a significant increase in both resting blood pressure (*p* < 0.01) and resting heart rate (*p* < 0,01) (pre-drug vs post-drug) (Table 1). No changes were observed in any respiratory variables (Table 1).

##### ***Role of A5 CNQX microinjections on the respiratory response to dlPAG stimulation***

The dlPAG stimulation after blockade of Non-NMDA receptors within the A5 with CNQX microinjection produced an increase in both respiratory rate (*p* < 0,001) and pleural pressure (*p* < 0,001) (post-drug vs post-drug stimulation), being the effect on the respiratory rate lower than at the pre-drug stimulation condition (*p* < 0,05) (Table 1).

It could be noted that after the microinjection of CNQX within the A5 region a significant lower amplitude of the respiratory response to dlPAG stimulation (*p* < 0,01) was observed compared with control situation (pre-drug vs pre-drug stimulations) (Fig. 5).

##### ***Role of A5 CNQX microinjections on the cardiovascular response to dlPAG stimulation***

An increase in both heart rate (*p* < 0,01) and blood pressure (*p* < 0,001) were evoked to dlPAG stimulation after CNQX microinjection (post-drug vs post-drug stimulation), being the effect on blood pressure higher than at pre-drug stimulation situation (*p* < 0,01) (Table 1).

On the other hand, the blockade of Non-NMDA receptors within the A5 reduced the amplitude of the cardiovascular response, both blood pressure (*p* < 0,05) and heart rate (*p* < 0,001), evoked to dlPAG stimulations were reduced compared with control situation (pre-drug vs pre-drug stimulation) (Fig. 5).

##### ***Other results***

Two animals were microinjected outside the study area. In one of them, a slight decrease in the cardiorespiratory response to dlPAG stimulation compare wth control situation was observed. The location of the microinjection was between the areas of Pr5VL-7n. In the other animal, there was no change between the two responses to dlPAG stimulation. The location of the microinjection was in the area of the Pr5VL and dorsomedial side (Pr5DM).

#### Metabotropic non-NMDA antagonists

##### *Cardiorespiratory effect produced by metabotropic glutamatergic antagonist in the A5 region: MCPG microinjection*

The blockade of A5 metabotropic glutamate receptors with MCPG (*n* = 7) did not produced significant changes in the cardiorespiratory variables at rest (pre-drug vs post-drug) (Table 1).

##### *Role of A5 MCPG microinjections on the respiratory response to dlPAG stimulation*

The respiratory response evoked to dlPAG stimulation after MCPG microinjection elicited an increase in both respiratory rate (*p* < 0,01) and pleural pressure (*p* < 0,001), which was similar to the effect of dlPAG stimulation alone (Table 1).

MCPG microinjected within the A5 region did not produce any significant changes in the amplitude of the respiratory response evoked to dlPAG stimulation compared with control situation (pre-drug vs pre-drug stimulation) (Fig. 5).

##### ***Role of A5 MCPG microinjections on the cardiovascular response to dlPAG stimulation***

After MCPG microinjection, a significant increase in both blood pressure (*p* < 0,001) and heart rate (*p* < 0,01) were elicited to dlPAG stimulation (post-drug vs post-drug stimulation) (Table 1). This response was similar to that observed after dlPAG stimulation alone (pre-drug stimulation) (Table 1).

Thus, the amplitude of both blood pressure and heart rate responses to dlPAG stimulation after MCPG microinjection was significantly lower compared with control situation (*p* < 0,05 and *p* < 0.001 respectively) (pre-drug vs pre-drug stimulation) (Fig. 5).

##### *Other results*

One animal was microinjected outside the study area. The microinjection did not produce any cardiorespiratory changes on its own or after stimulation of the dlPAG compared with control situation. The microinjection was located between the areas of Pr5VL, Sp5 and 7n).

## Discussion

The results demonstrate the presence of a close functional relationship between the mesencephalic dlPAG and the pontine A5 region and give a key role for glutamate in this interaction.

We have corroborated the conclusions drawn from our previous study [19], where we showed that the A5 pontine region modulates the heart and respiratory rate components of the cardiorespiratory response evoked by dlPAG stimulation. In addition, our new data add a new insight into this modulation. Thus, we can state that one of the neurotransmitters responsible for the changes in heart rate and respiratory rate at the level of the A5 region is glutamate. Furthermore, in the second phase of this study, we focused on which of the different families of glutamate receptors were involved in these interactions.

The results indicate that non-NMDA receptors seem to be responsible for the modulation of the respiratory response, while the modulation of the heart rate response evoked by dlPAG stimulation seems to be more distributed across the different glutamate receptor families, being the NMDA receptor the most important contributor to this modulation.

### Methodological considerations

Previously we tried to stimulate dlPAG chemically as other authors [12]**.** In order to stimulate dlPAG we used bicuculline, but we did not obtain similar results in control responses to repeated dlPAG microinjections. For that reason, we decided to use electrical stimulation. Authors are aware of the limitations of using electrical stimulation techniques, in which both somatas of the neurons located within the dlPAG and the passing axons from other areas are stimulated, we decided to use this technique in order to compare the results with those of previous studies [19, 20]. Even so, it is clear that it would be very interesting to be able to extend this type of study in the future by means of optogenetic techniques to stimulate or inhibit the dlPAG combining with blocking the A5 region via both muscimol microinjections (general neurotransmission) and kynurenic acid microinjections (glutamatergic neurotransmission).

We attempted bilateral microinjections of kynurenic acid in an initial set of 5 animals, but they produced severe and progressive cardiorespiratory depression leading to death of the animals (data not included in the study). Therefore, neuropharmacological blockade of glutamate receptors with the different study drugs was performed unilaterally on the right hemisphere. Both the doses and volumes of the microinjections administered were selected according to observations from similar studies [7, 20].

### Cardiorespiratory responses to dlPAG stimulations after A5 region microinjections

Stimulation of cell bodies of the A5 region evoked a cardiovascular response that resembles to that obtained by electrical stimulation from different ¨defence regions¨ (DMH-PeF and dlPAG) [5]. Furthermore, as our group have previously reported, the A5 region seems to have a key role in the modulation of the cardiorespiratory response evoked from DMH-PeF or dlPAG [19, 21]. Namely, in our latest work [19], we demonstrated a functional connections between the dlPAG and the A5 region due to a decrease in heart rate and respiratory rate responses to dlPAG electrical stimulation when cell bodies of the A5 region were inhibited with muscimol. We also showed, using electrophysiological extracellular recordings, that several putative A5 cells modified mono or polysinaptically their activity during dlPAG stimulation. All these data suggest that neurons of the A5 region could modulate the cardiorespiratory response elicited from dlPAG both, through a direct or an indirect pathway.

It is known that dlPAG activation exerts an increase in sympathetic tone, including an increase in blood pressure, heart rate, respiratory rate and skeletal muscle vasodilation. All of these actions are included in the classical fight and flight or ´defence response´ that also generates active defensive actions such as jumping and running [17, 19]. Glutamatergic neurons within the dlPAG are the latest responsible of this activation [8].

The A5 region has a rather wide distribution of all glutamate receptor subtypes, either ionotropic NMDA (NR1-NR2D subunits) [10], or non-NMDA (AMPA and kainate) [25, 35] and metabotropic (mGluR I, II and III) [29]. For that, a set of experiments were performed with the purpose of finding out what could be the possible role of glutamatergic neurotransmission in the A5 region on the cardiorespiratory response to dlPAG stimulation. For this purpose, kynurenic acid, MK-801, CNQX and MCPG were microinjected within the A5 region. Chiefly, we have focussed on the effects of the different antagonists on resting cardiorespiratory parameters, to end with the effects on the cardiorespiratory response evoked to dlPAG stimulation.

### Resting conditions

As we have observed in previous studies [20, 21] blocking the general neurotransmission within the A5 region, the nonspecific blockade of glutamate receptors within the A5 region produced a significant decrease in respiratory rate due to an increase in expiratory time. However, a slight but non-significant decrease in the resting respiratory rate were observed when A5 region glutamate receptors were blocked with DAP5, CNQX or MCPG. It seems that it is necessary to block all the different families of glutamate receptors to obtain a significant effect on the resting respiratory rate.

This observation is consistent with previous works speculating on a chemoreceptor role for the A5 region due to its connectivity with the retrotrapezoid nucleus (RTN), a nucleus that is involved in chemoreception. Therefore, the inhibition of these projections could lead to the decrease of respiratory rate at rest after inhibiting or lesioning the A5 region [26, 27].

### Effects on the cardiorespiratory response to dlPAG electrical stimulation

There is wide evidence in the scientific literature for a sympathoexcitatory role of the dlPAG, characterised by tachypnoea, tachycardia and hypertension [7, 19, 20]. This cardiorespiratory response is elicited due to a direct activation to the rostral ventrolateral medulla (RVLM) neurons which, in turn, activate sympathetic preganglionic neurons in the intermedio lateral cell column of the spinal cord (IML), producing the pressor and tachycardic effect [18].

Other studies have shown the modulation of RVLM neurons by polysinaptic projections from the dlPAG [3]. They also suggest the existence of these two separated cardiorespiratory pathways and described that an indirect projection from the dlPAG to the medulla via interactions with DMH-PeF, parabrachial complex and cuneiform nucleus facilitated the associated sympathoexcitatory cardiorespiratory changes evoked during dlPAG stimulation.

The cardiorespiratory role of the A5 region has also been widely studied. We have previously shown that its activation by glutamate microinjection produces a cardiovascular response very similar to that of the dlPAG [5]. Moreover, in a recent work, we have shown its role in modulating the cardiorespiratory response evoked by dlPAG [19], confirming the A5 region partially mediates the cardiorespiratory response evoked from the dlPAG. This is interesting because it re-emphasises the existence of two independent pathways for the control of the pressor and tachycardic response, confirming both the existence of the direct and indirect connections of dlPAG with other central nuclei involved in the control of the pressor response, as well as the essential role of the A5 region in the regulation of the heart rate component of the baroreceptor reflex.

At this point, the data obtained in this study have shown that the general blockade of glutamate receptors by kynurenic acid reduces the tachycardic response (almost abolished) and the tachypnoea evoked by electrical stimulation of dlPAG. The pressor response is unchanged. Thus, we demonstrate that activation of the A5 region is critical to prevent the expression of the heart rate component resulting from baroreceptor reflex activation [23]. The decrease of the respiratory response, and the slight non-significant decrease in the pressor response also seem to corroborate the chemoreceptor role of this area [13], giving a role to the glutamatergic neurotransmission in those effects.

Having achieved the first objective of this study, confirming that glutamate receptors in the A5 region mediate the cardiorespiratory response evoked by dlPAG, we set the second objective, to elucidate the specific role of ionotropic (NMDA and non-NMDA) and metabotropic receptors in this modulation. Specifically, all microinjected drugs broadly reduce the heart rate component and attenuate to a lesser extent the pressor response evoked by electrical stimulation of the dlPAG. Thus, our results suggest that the cardiovascular response to dlPAG stimulation is modulated by a broad activation of the different families of glutamate receptors in the A5 region, and that the effect is depending on the distribution of these receptor subtypes in the A5 region. Regarding to the respiratory response, only the non-NMDA receptor antagonist (CNQX) is able to reduce the tachypnoea evoked by electrical stimulation of dlPAG, which indicates that this family of A5 glutamate receptors seems to be responsible for modulating the respiratory responses.

### Final consideration

The data provided by our study demonstrate for the first time that A5 region glutamate receptors are involved in the cardiorespiratory response evoked during dlPAG electrical stimulation.

The decrease or abolition of the tachycardia evoked by electrical stimulation of the dlPAG following blockade of the different glutamate receptor subtypes in the A5 region highlights the role of glutamate in this pontine region, confirming the importance of its integrity in interfering with the full expression of the baroreceptor reflex [21].

The study must be extended to contribute to clarify the importance of the integrated activity of all subtypes of glutamate receptors within the A5 region on the control of autonomic functions and its clinical physiopathology.

## Data Availability

The data included in the study are available in a generalist data repository provided by the University of Málaga (https://riuma.uma.es/xmlui/).
